# Differential microRNA Expression in Newcastle Disease Virus-Infected HeLa Cells and Its Role in Regulating Virus Replication

**DOI:** 10.3389/fonc.2021.616809

**Published:** 2021-06-04

**Authors:** Yu Chen, Shanshan Zhu, Yuru Pei, Jiao Hu, Zenglei Hu, Xiaowen Liu, Xiaoquan Wang, Min Gu, Shunlin Hu, Xiufan Liu

**Affiliations:** ^1^Animal Infectious Disease Laboratory, College of Veterinary Medicine, Yangzhou University, Yangzhou, China; ^2^Jiangsu Co-innovation Center for Prevention and Control of Important Animal Infectious Diseases and Zoonosis, Yangzhou University, Yangzhou, China; ^3^Jiangsu Key Laboratory of Zoonosis, Yangzhou University, Yangzhou, China

**Keywords:** NDV, oncologic virus, miRNA expression profile, HeLa cells, hsa-miR-4521, FAM129A

## Abstract

As an oncolytic virus, Newcastle disease virus (NDV) can specifically kill tumor cells and has been tested as an attractive oncolytic agent for cancer virotherapy. Virus infection can trigger the changes of the cellular microRNA (miRNA) expression profile, which can greatly influence viral replication and pathogenesis. However, the interplay between NDV replication and cellular miRNA expression in tumor cells is still largely unknown. In the present study, we compared the profiles of cellular miRNAs in uninfected and NDV-infected HeLa cells by small RNA deep sequencing. Here we report that NDV infection in HeLa cells significantly changed the levels of 40 miRNAs at 6 h post-infection (hpi) and 62 miRNAs at 12 hpi. Among 23 highly differentially expressed miRNAs, NDV infection greatly promoted the levels of 3 miRNAs and suppressed the levels of 20 miRNAs at both time points. These 23 miRNAs are predicted to target various genes involved in virus replication and antiviral immunity such as ErbB, Jak-STAT, NF-kB and RIG-I-like receptor. Verification of deep sequencing results by quantitative RT-PCR showed that 9 out of 10 randomly selected miRNAs chosen from this 23-miRNA pool were consistent with deep sequencing data, including 6 down-regulated and 3 up-regulated. Further functional research revealed that hsa-miR-4521, a constituent in this 23-miRNA pool, inhibited NDV replication in HeLa cells. Moreover, dual-luciferase and gene expression array uncovered that the member A of family with sequence similarity 129 (FAM129A) was directly targeted by hsa-miR-4521 and positively regulated NDV replication in HeLa cells, indicating that hsa-miR-4521 may regulate NDV replication *via* interaction with FAM129A. To our knowledge, this is the first report of the dynamic cellular miRNA expression profile in tumor cells after NDV infection and may provide a valuable basis for further investigation on the roles of miRNAs in NDV-mediated oncolysis.

## Introduction

Newcastle disease virus (NDV) belongs to the genus *orthoavulavirus* within the subfamily *Avulavirinae* of the family *Paramyxoviridae* (1). The genome of NDV is composed of a non-segmented, negative-sense, and single-stranded RNA, encoding six different proteins: nucleocapsid protein (NP), phosphoprotein (P), matrix protein (M), fusion protein (F), haemagglutinin-neuraminidase (HN), and RNA-dependent RNA polymerase (L) (2). In addition to infecting more than 250 bird species (3), NDV preferentially replicates in and exhibits a strong cytotoxic potential against different human tumor cells (4, 5). The first report about the anti-neoplastic activity of NDV appeared about 50 years ago (6). Since then, many observations made in animal tumor models as well as in cancer patients either *in vitro* or *in vivo*, have demonstrated the special anticancer properties of NDV (7–9). Several mechanisms for the oncolytic activity of NDV have also been identified: first, a direct extrinsic and intrinsic apoptosis following NDV infection due to the defective interferon signaling pathways in tumor cells (10–12) and second, an indirect effect through recruitment of the innate and adaptive arms of the host immune system (13, 14). On account of these superior characteristics, NDV has been tested as a novel oncolytic agent for cancer virotherapy.

MicroRNAs (miRANs), a class of highly conserved small non-coding RNAs with approximately 21-23 nucleotides long, can directly bind to the 3′-untranslated region (UTR) of the mRNA to suppress protein synthesis (15–17). Because of its role in regulating protein synthesis, miRNA has been implicated in regulating cell growth, differentiation, development, apoptosis, and oncogenesis (18–20). Many viruses can regulate miRNA expression, whereas altered miRNA expression in turn affects virus replication and their pathogenesis (21). Emerging evidence demonstrates that miRNAs are also involved in the tumor lysis induced by oncolytic viruses such as measles virus (MV) (22, 23) and oncolytic adenovirus (24). miR-31 and miR-128 inhibit the expression of MV receptor poliovirus receptor-related 4 (PVRL4) by direct binding to the 3’UTR of PVRL4 mRNA, thus suppressing MV infection in glioblastoma cells (22, 23). Otherwise, MV induces miR-31 expression, leading to the downregulation of PVRL4 and preventing virus entry into the host cells (22). Oncolytic adenovirus infection leads to the differential expression of thirty-three miRNAs in prostate cancer cells, including 5 upregulated and 29 downregulated (24). Moreover, among these differentially expressed miRNAs, miR-26b promotes adenovirus-induced cell death, viral progeny release, and propagation in prostate cancer cells, which indicating that miR-26b in combination with oncolytic adenoviruses may achieve synergistic antitumor activity (24). These observations collectively suggest that host miRNAs play an important role in oncolytic virus replication in tumor cells.

Although the important roles of host miRNAs played in oncolytic virus infection have been extensively studied, the role of miRNAs in NDV oncolytic process is still poorly understood. Our present study focuses on the expression and function of miRNAs in NDV-infected HeLa cells. We report here that NDV infection changed the landscape of miRNA expression and identified hsa-miR-4521 regulates NDV replication *via* interaction with the member A of family with sequence similarity 129 (FAM129A). Our study highlights the importance of miRNA expression in regulating NDV replication and its oncolytic activity and may pave the way to understand the oncolytic characteristics of NDV and the mechanisms of virus-host interactions.

## Materials and Methods

### Virus and Cells

HeLa cells (ATCC CCL-2) and DF-1 cells (ATCC CRL-12203) were cultured in Dulbecco’s modified Eagle’s medium (DMEM) (Life Technologies, USA) supplemented with 10% fetal bovine serum (FBS) (Life Technologies, USA), 100 U/mL penicillin (Invitrogen, USA) and 100 μg/mL streptomycin (Invitrogen, USA) at 37°C under 5% CO_2_ atmosphere. Velogenic NDV strain Herts/33 (Accession Number: AY741404) was obtained from Dr. D. J. Alexander (Animal Health and Veterinary Laboratories Agency, UK) and maintained in our laboratory.

### Viral Infection and RNA Isolation

HeLa cells were infected with NDV strain Herts/33 at a multiplicity of infection (MOI) of 1 for 1h in serum-free DMEM. Then, the cells were washed three times with phosphate-buffered saline (PBS) and incubated at 37°C in DMEM supplemented with 2% FBS. NDV-infected HeLa cells were trypsinized with 0.5 mL of trypsin (2μg/mL) (Sigma, USA) at 6 and 12 h post-infection (hpi) respectively and centrifuged at 1200g for 8 min and washed three times with ice-cold PBS. Sham infected cells were used as the mock group and were collected as the same way as NDV-infected cells. Three replicates of each group were prepared and pooled separately for subsequent total RNA extraction using the EasyPure RNA kit (TransGen Biotech, China) according to the manufacturer’s instructions. The total concentration and purity of total RNA samples were measured with a Nano Drop ND-2000 spectrophotometer (Thermo Fisher Scientific, USA) and an Agilent 2200 Bioanalyzer (Agilent Technologies, USA).

### Library Construction and Small RNA (sRNA) Deep Sequencing

The total RNA from each sample was sequentially ligated to 3’ and 5’ sRNA adapters. Then, cDNA was synthesized and amplified using TruSeq sRNA Sample Preparation Kit (Illumina, USA) according to the manufacturer’s instructions. 145–160 bp PCR-amplified fragments were extracted and purified from the PAGE gel. The DNA fragments in the eligible libraries were eventually used for sequencing on an Illumina HiSeq 2500 instrument (Illumia Inc., USA) according to the manufacturer’s instructions.

### Analysis of Deep Sequencing Data

All raw sequencing data was processed using the Short Oligonucleotide Alignment Program (SOAP) software (BGI Company, China) (25) to obtain clean reads from each library as follows: except for low quality reads, reads with 5′ primer contaminants, reads without a 3′ primer, reads with no insert tags, reads with poly A tags, reads shorter than 18 nt and longer than 30 nt. After quantity control program, the clean reads were mapped to Homo sapiens genome using Burrows-Wheeler Alignment Tool (BWA) (26) and their expression and distribution patterns were analyzed using the SOAP software. The clean reads were mapped against NCBI Genebank (http://www.ncbi.nlm.nih.gov/blast/Blast.cgi/), Rfam database (http://www.sanger.ac.uk/Software/Rfam/) and miRBase version 21.0 database (http://www.mirbase.org/) to annotate sRNAs, including miRNAs, rRNAs, tRNAs, snRNAs, snoRNAs, piRNAs, scRNAs, srpRNAs, repeats, exon and intron sequences. The quantity of known miRNAs is normalized and showed as reads per million (RPM) using the following computational formula: RPM= (actual miRNA count/Total number of clean reads) ×10^6^. The R package *DESeq* was used to detect the differential expression of the known miRNAs between different groups (27). A *p*-value < 0.05 and│log2 (fold change) │>1 were set as the default thresholds for significantly differential expression.

### Target Prediction and Functional Enrichment of Differentially Expressed miRNAs

Targetscan (http://www.targetscan.org/) and miRanda (https://www.microrna.org/) were used to predict the target genes of each differentially expressed miRNA at default settings. The functions of predicted target genes were evaluated using GO and KEGG enrichment analysis. GO and KEGG analyses were carried out using the R package named Cluster Profiler based on hyper-geometric distribution. The *p-*value was calculated through Fisher Exact Test and corrected by false discovery rate (FDR). GO terms and KEGG pathways with corrected *p*-value < 0.05 was considered to represent significant enrichment.

### Plasmid Construction

The 3’ UTR of FAM129A containing the hsa-miR-4521 binding sites was first amplified from total RNA of HeLa cells with specific primers (Forward: 5’-CTCGAGGCAGTCCTAGCTACTTGGGTG-3’; Reverse: 5’- GCGGCCGCGGCTTTGCCCATAGGAGAGAT-3’) and then cloned into the *Xho*I/*Not*I restriction sites in the pmiR-RB-reporterTM vector (Ribobio, China) to generate pmiR-FAM129A-3’UTR-wild plasmid. pmiR-RB-reporter TM vector contains two promoters and can simultaneously express renilla luciferase and firefly luciferase—the renilla luciferase is used for evaluating miRNA regulation, and firefly luciferase is used for the normalization of transfection efficiency. To construct a hsa-miR-4521 binding sites-mutated reporter vector (pmiR-FAM129A-3’UTR-mut), mutation was achieved by changing the hsa-miR-4521 binding seed sequences from TCCTTAG to TGGAATG using Fast Mutagenesis System kit (Transgen Biotech, China) according to manufacturer’s instructions. Primers used for mutation are as follows: Forward: 5’- AACAGCATTTTTTTGGAAAGCCTCTGT -3’; Reverse: 5’- TTCCAAAAAAATGCTGTTCATTTAGATCAG-3’. An FAM129A overexpression construct was generated by amplifying the FAM129A coding sequence with a pair of specific primers (Forword:5’- GGATTCATGGGCGGCTCAGCCTC-3’; Reverse:5’- AAGCTTTCACTCCTCTGAGGGCAGC-3’), and it was subsequently cloned into the *BamH*I/*Hind* III restriction sites in the overexpression plasmid vector pCMV-Blank (Beyotime, China). All primers used for plasmids construction were synthesized by Sangon Company, China. All constructs were verified by sequencing.

### MiRNA Oligonucleotides and Small Interference RNA (siRNA) Transfection

HeLa cells with a density of 60% confluence were transfected with indicated miRNA oligonucleotides, including miRNA mimics or inhibitors or negative control, or FAM129A specific siRNA (siRNA-FAM129A) or siRNA negative control using Lipofectamine 3000 Reagent (Life Technologies, USA), in accordance with the manufacturer’s recommended protocol. The siRNAs sequences are as follows: siRNA-FAM129A: 5’-CCACGAGGCTGCTGACCAGTT-3’ (sense), 5’-CTGGTCAGCAGCCTCGTGGTT-3’ (anti-sense); siRNA-NC: 5’-TTCTCCGAACGTGTCACGTTT -3’ (sense), 5’-ACGTGACACGTTCGGAGAATT -3’ (anti-sense). MiRNA oligonucleotides and siRNAs were designed and synthesized by Genepharma Company, China and transfected at a final concentration of 100 nM (miRNA oligonucleotides) or 20 nM (siRNA).

### Stem-Loop qRT-PCR of miRNAs

MiRNA Extraction Kit (HaiGene, China) was used to extract miRNAs from HeLa cells according to manufacturer’s instructions. Then, cDNA synthesis was carried out according to the protocol of One Step miRNA cDNA Synthesis Kit (HaiGene, China), in which poly(A) tailing of the miRNAs is followed by reverse transcription with a tagged poly(T) primer described by Balcells et al. (28). Quantification of miRNA was performed by HG miRNA SYBR Green PCR Kit (HaiGene, China) under the following conditions: 95°C for 15 min, 35 cycles of 95°C for 5s, and 60°C for 30s in LightCycler 480 (Roche, Switzerland). Primers used to detect miRNA expression are presented in **Table 1**. MiRNA expression levels were normalized to U6 small nuclear RNA levels using the 2^-ΔΔCt^ method.

**Table 1:** Primers for analysis of miRNA expression by qRT-PCR.

Primer name	Forward primer (5’-3’)	Reverse primers (5’-3’)
Reverse transcription	GTCGGTGTCGTGGAGTCGTTTGCAATTGCACTGGATTTTTTTTTTTTTTTTTT	
hsa-miR-4521	GCTAAGGAAGTCCTGTGCT	GTCCAGTTTTTTTTTTTTTTTCTGAG
hsa-miR-375	AGTTTGTTCGTTCGGCTC	GGTCCAGTTTTTTTTTTTTTTTCAC
hsa-miR-1260b	GATCCCACCACTGCCA	GGTCCAGTTTTTTTTTTTTTTTATGG
hsa-miR-877-5p	GTAGAGGAGATGGCGCA	GTCCAGTTTTTTTTTTTTTTTCCCT
hsa-miR-5010-3p	GCAGTTTTGTGTCTCCCATTC	GGTCCAGTTTTTTTTTTTTTTTCTG
hsa-miR-7641	GCAGTTGATCTCGGAAGCTA	GGTCCAGTTTTTTTTTTTTTTTGCT
hsa-miR-1277-5p	GCAGCGCAGAAATATATATATATATGTACG	GGTCCAGTTTTTTTTTTTTTTTATACG
hsa-miR-3128	CAGTCTGGCAAGTAAAAAACTCT	GGTCCAGTTTTTTTTTTTTTTTATGAGA
hsa-miR-6818-3p	AGTTGTCTCTTGTTCCTCACA	GGTCCAGTTTTTTTTTTTTTTTCTGT
U6	CTCGCTTCGGCAGCACAT	GGAACGCTTCACGAATTTGCG

### Detection of Viral Growth Curve

HeLa cells transfected with indicated miRNA oligonucleotides or siRNAs or overexpression plasmids or mock-treated were infected with NDV strain Herts/33 at an MOI of 1. After 1h of incubation, the supernatant was discarded and the infected cells were replenished with DMEM medium containing 1% FBS. The culture supernatants were collected and replaced with an equal volume of fresh media at different time points post infection. The viral contents in the supernatants were quantified by the 50% tissue culture infectious dose (TCID_50_) in DF-1 cells.

### Dual Luciferase Assay

HeLa cells were seeded at a density of 5 × 10^4^ cells per well in 24-well plates. After 24h, the cells were co-transfected with 500ng pmiR-FAM129A-3’UTR-wild or pmiR-FAM129A-3’UTR-mut plasmids and 100nM hsa-miR-4521 mimics or mimics negative control (mimics NC) or inhibitor or inhibitor negative control (inhibitor NC) using the Lipofectamine 3000 Reagent. Forty-eight hours after transfection, luciferase assays were measured using Dual-GLO^®^ Luciferase Assay System Kits (Promega, USA) following the manufacturer’s instructions by a Fluorescence/Multi-Detection Microplate Reader (Biotek, USA). The relative luciferase activity was calculated by calculating the ratio of renilla luciferase activity to firefly luciferase activity.

### qRT-PCR and Western Blot Assay

To detect FAM129A expression level, HeLa cells in 12-well plates were transfected with hsa-miR-4521 mimics or mimics NC or inhibitor or inhibitor NC or mock-treated. Eighteen hours post transfection, the total RNAs were extracted from the cells using TRIzol (TransGen Biotech, China) following the manufacturer’s instructions. The cDNA synthesis and qRT-PCR reaction were performed using TransScript Green One-Step qRT-PCR Super Mix (TransGen Biotech, China) according to the manufacturer’s instructions. The relative expression of FAM129A was normalized with glyceraldehyde phosphate dehydrogenase (GAPDH) and calculated using the 2^-ΔΔCT^ method. QRT-PCR experiments were performed in LightCycler 480 (Roche, Switzerland) and primers sequences are as follows: FAM129A forward: 5’-GCTCATGGGACCAGTGAGCT-3’; FAM129A reverse: 5’-CGGAATGCAGCGGAAGATTC-3’; GAPDH forward: 5’-GGCAAAGTCCAAGTGGTGGC-3’; GAPDH reverse: 5’-GCACCTGCATCTGCCCATTT-3’. To detect FAM129A expression at protein level, cell lysates were obtained with RIPA lysis buffer, and proteins were analyzed by western blot. β-actin serves as a control. The following antibodies were used: Mouse polyclonal anti-FAM129A (Sangon Biotech, China), mouse monoclonal anti-β-actin (Sangon Biotech, China) and HRP-conjugated Goat anti-Mouse IgG (Sangon Biotech, China). The relative protein quantification was analyzed by Image J software (Bethesda, USA)

### Statistical Analysis

All experiments were performed in triplicate. Statistical significance was determined using Student’s t-test by GraphPad Prism 5 (GraphPad Software, San Diego, USA). Data are presented as the means ± SD. *P*-value <0.05 was considered as a significant difference.

## Results

### Confirmation of Velogenic NDV Strain Herts/33 Infection in HeLa Cells

**Figure 1:**
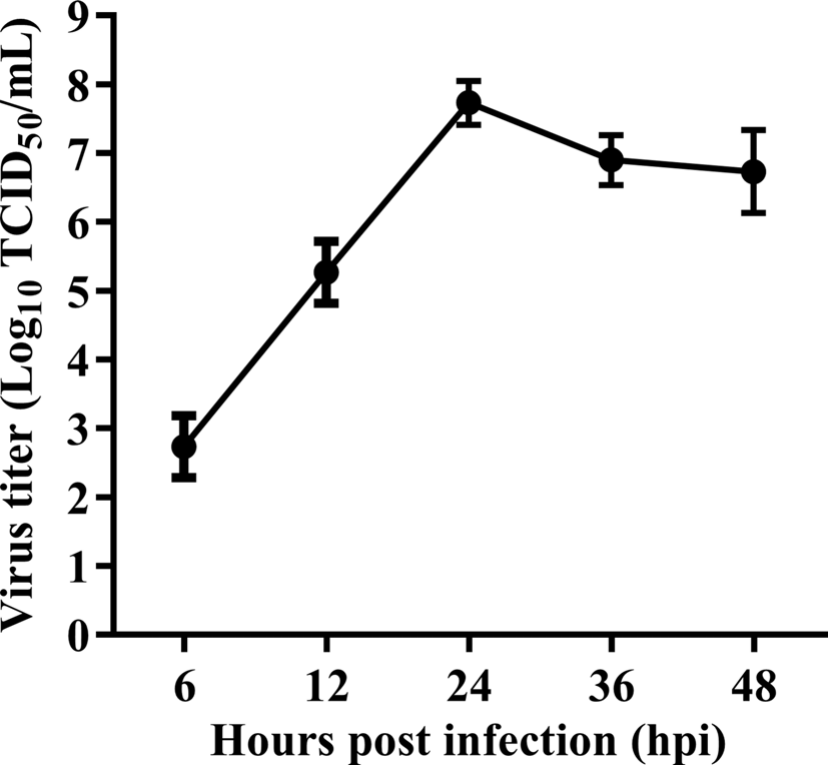
Replication kinetic of velogenic NDV strain Herts/33 in HeLa cells. HeLa cells were infected with velogenic NDV strain Herts/33 at an MOI of 1. Subsequently, the cellular supernatants were taken at indicated time points and titrated by TCID_50_ assay in DF-1 cells. Data are from three independent experiments and presented as the means ± SD.

The oncolytic activity of different NDV strains including pathogenic (Ulster and PV701) and non-pathogenic (La Sota and HUJ) strains have already been studied in several tumor cell lines (10, 29–31). We first evaluated the ability of velogenic NDV strain Herts/33 to replicate in HeLa cells, a human cervical cancer cell line. As shown in **Figure 1**, the virus replicated rapidly in HeLa cells inoculated with 1 MOI of the Herts/33 NDV strain. Virus titers peaked at 24 hpi, which reached 7.55 x10^7^ TCID_50_/mL.

### Overview of the Deep Sequencing Data

**Figure 2:**
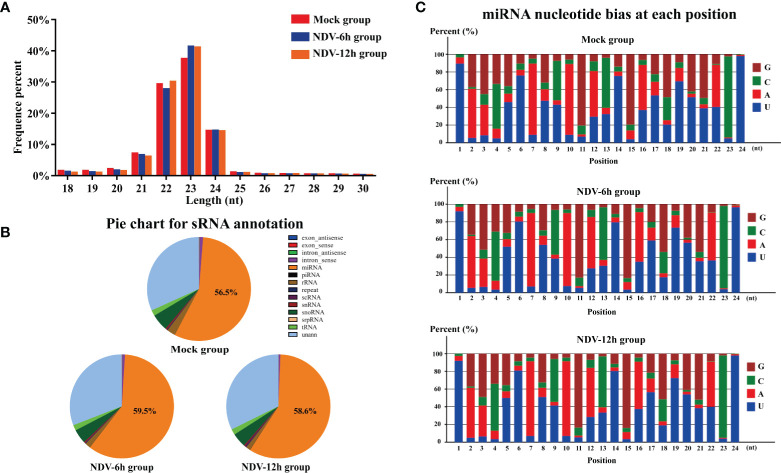
Analysis of sRNA data of each groups. **(A)** Length distributions of the clean reads from each group. **(B)** Annotation of the clean reads for each group showing the quantity of sRNA components. **(C)** Analysis of the first nucleotide bias in the identified known miRNAs of each group.

We next determined if NDV affected cellular miRNAs expression. Three sRNA libraries constructed from uninfected HeLa cells or the cells infected with NDV at 6 and 12 hpi were sequenced using an Illumina platform. RNA-seq data have been deposited in the ArrayExpress database at EMBL-EBI under accession number E-MTAB-9690 (https://www.ebi.ac.uk/arrayexpress/experiments/E-MTAB-9690). The deep sequencing data were summarized in **Table 2**. After quality control procedures, 10868309, 10485342 and 12224749 clean reads of mock, NDV-6h and NDV-12h, respectively, were further analyzed. Approximately 90% clean reads from all three groups were 21-24 nt, and most sRNAs were 23nt (**Figure 2A**), which was consistent with the typical size of miRNA from Dicer-derived products. The distribution of sRNAs is shown in **Figure 2B**. In general, more than 55% of clean reads were identified as miRNAs in each group. Known miRNAs in each group were identified by alignment clean reads to the relevant bioinformatics software, as described in the *Materials and Methods*. 189, 193 and 192 miRNAs were identified in mock, NDV-6h and NDV-12h, respectively. Analysis of the first nucleotide bias for miRNAs showed Uridine (U) dominated the first position, which is a characteristic feature of mature miRNAs (**Figure 2C**).

**Table 2:** Overview of the deep sequencing data of each group.

**Sample**	**Total reads**	**High quality**	**3’adapter**	**Insert**	**5’adapter**	**>18nt**	**PolyA**	**Clean reads**
**Mock**	11575450	11481503 (99.19%)	265325 (2.31%)	15732 (0.14%)	4316 (0.04%)	327229 (2.85%)	592 (0.01%)	10868309 (94.66%)
**NDV-6h**	11109386	11022881 (99.22%)	202384 (1.84%)	6666 (0.06%)	3102 (0.03%)	324944 (2.95%)	443 (0.00%)	10485342 (95.12%)
**NDV-12h**	12825954	12725585 (99.22%)	261229 (2.05%)	13227 (0.10%)	4642 (0.04%)	221147 (1.74%)	591 (0.00%)	12224749 (96.06%)

### Differential Expression of miRNAs in NDV-Infected and -Uninfected HeLa Cells

**Figure 3:**
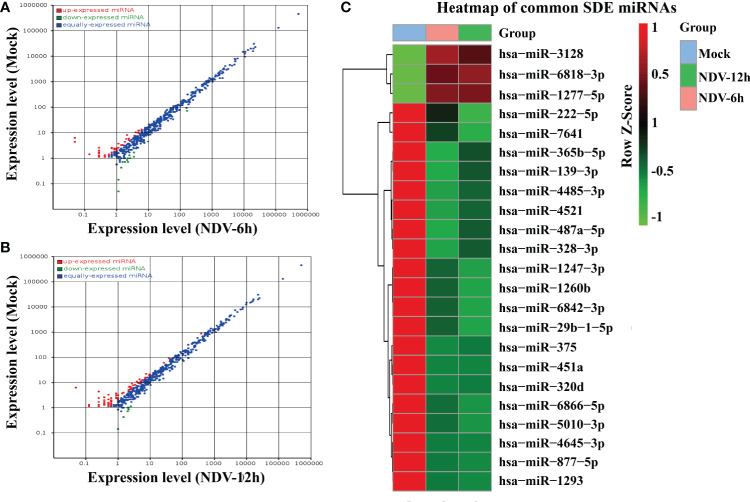
Comparison of the SDE miRNAs between NDV-infected and -uninfected HeLa cells. The scatter plot of SDE miRNAs of two comparable groups: **(A)** NDV-6h *vs.* Mock and **(B)** NDV-12h *vs.* Mock. **(C)** The heat map of 23 common SDE miRNAs in two comparable groups. The expression index with the ROW Z-Score is on the right representing degree of abundance in each group. Red shaded areas indicate higher expression, whereas green shaded areas indicate lower expression and black denotes the relatively similar expression between NDV-infected and -uninfected groups.

The differentially expressed miRNAs between each group were identified through *DESeq* package. In total, 40 miRNAs of NDV-6h and 62 miRNAs of NDV-12h were significantly differentially expressed (SDE) when compared with those in uninfected controls, respectively. Among these SDE miRNAs, 23 miRNAs, including 3 up-regulated and 20 down-regulated, showed the same expression patterns in NDV-6h and NDV-12h. The scatter plot of miRNA expression profiles was shown in **Figure 3A** (NDV-6h VS Mock) and **Figure 3B** (NDV-12h VS Mock) and the hierarchical clustering heat map of 23 common SDE miRNAs was shown in **Figure 3C**.

### Target Genes Prediction and Functional Enrichment Analysis

**Figure 4:**
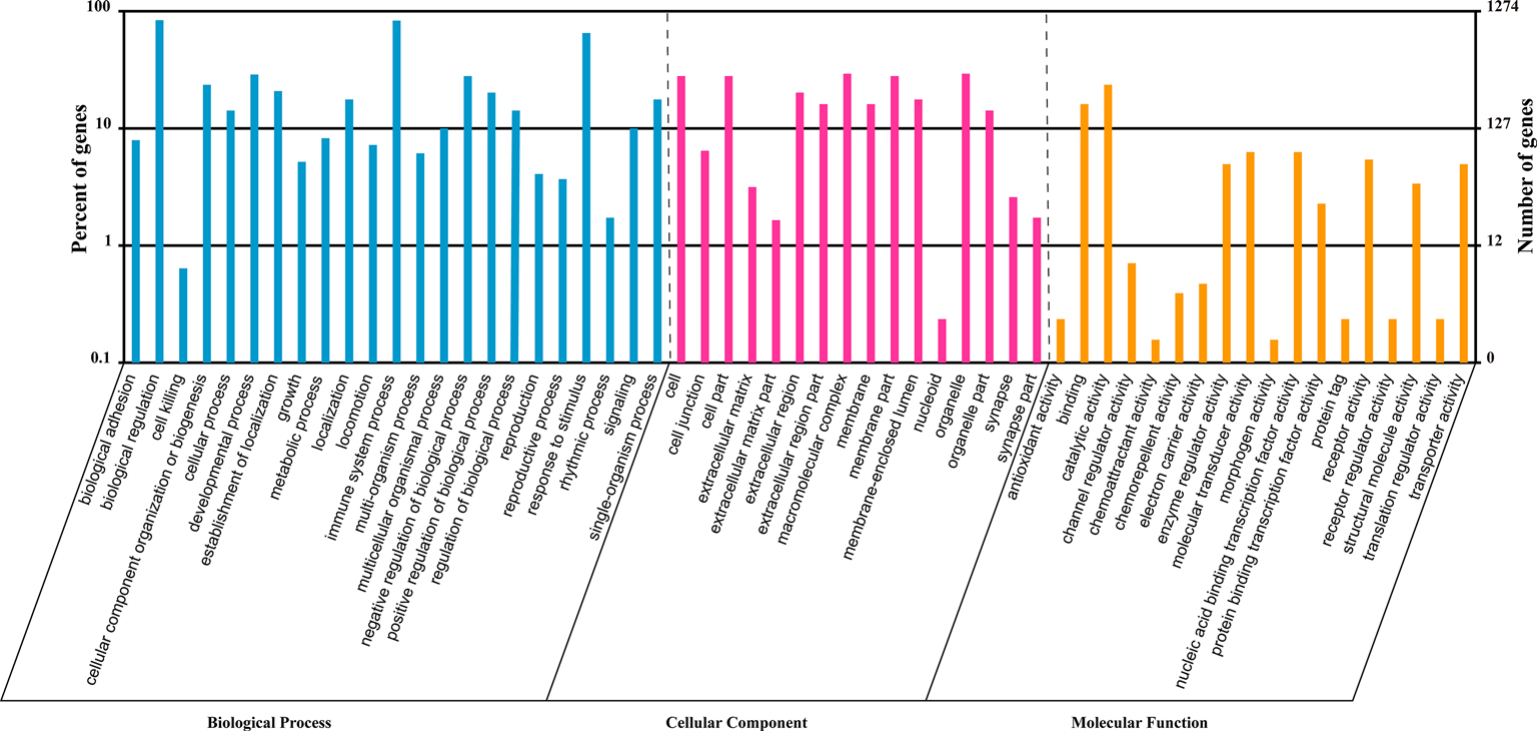
GO analysis of the predicted targets for the 23 common SDE miRNAs. GO analysis was performed by the R package named Cluster Profiler based on hyper-geometric distribution. The *P*-value was calculated through Fisher Exact Test and corrected by FDR. The corrected *P*-value<0.05 was considered as significant enrichment and showed in the result.

**Figure 5:**
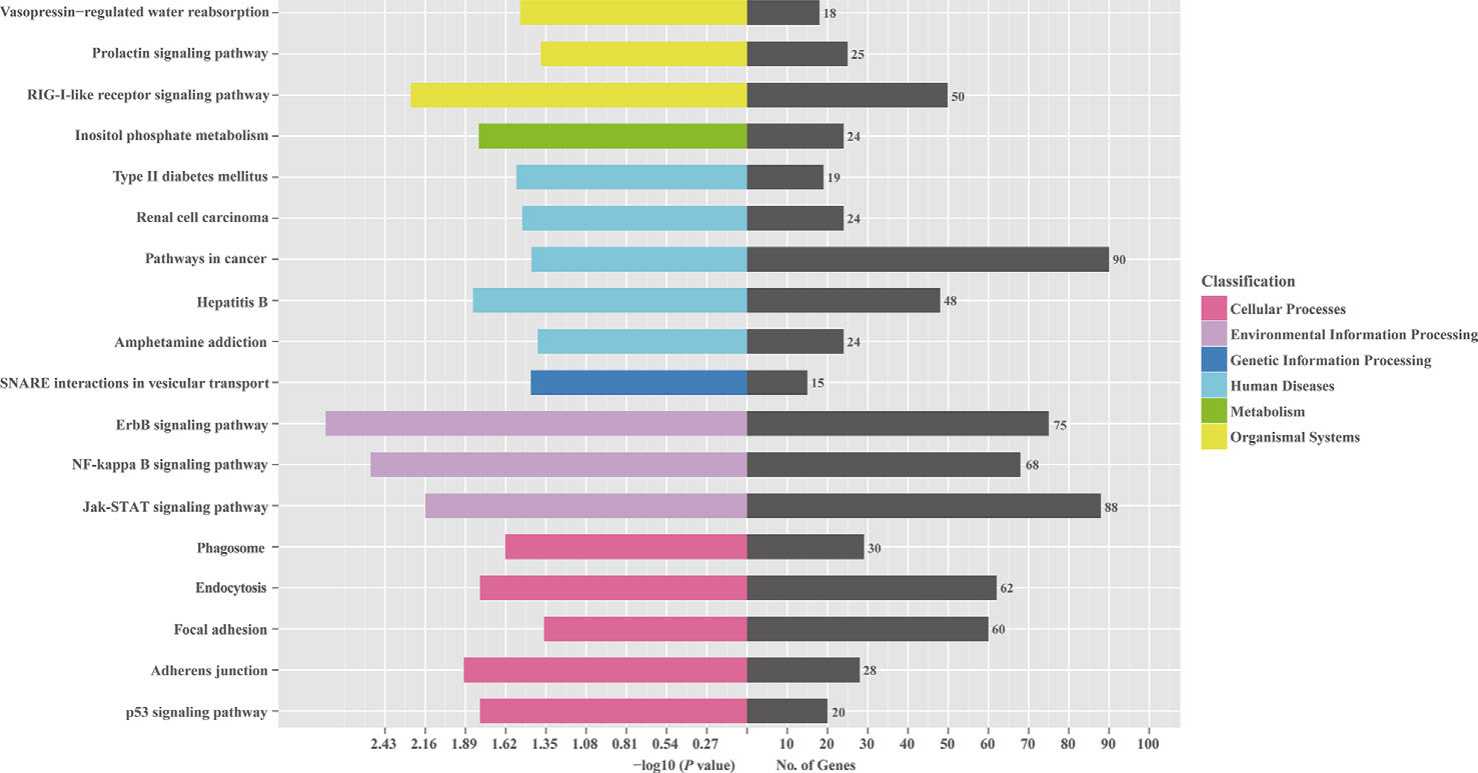
KEGG pathway analysis for the predicted target genes of 23 common SDE miRNAs. The predicted target genes of 23 common SDE miRNAs were chosen for KEGG pathway analysis using the R package named Cluster Profiler based on hyper-geometric distribution. The *P*-value was calculated through Fisher Exact Test and corrected by FDR. The corrected *P*-value<0.05 was considered as significant enrichment.

To investigate the role of the common SDE miRNAs, the potential target genes were predicted using two commonly-used miRNA target prediction programs: TargetScan and miRanda. In total, 43781 putative targets were found in the combined outputs of both programs. All the predicted targets were then subjected to GO and KEGG analyses. The GO analysis revealed that target genes were associated with biological regulation, immune system process, responses to stimulus and other cellular processes (**Figure 4**). KEGG pathway enrichment was shown in **Figure 5**. The results revealed that target genes were involved in numerous biological processes, such as ErbB signaling pathway, JAK-STAT signaling pathway, NF-kappa B signaling pathway, RIG-I-like receptor signaling pathway, etc.

### Validation of Deep Sequencing Results by qRT-PCR

**Figure 6:**
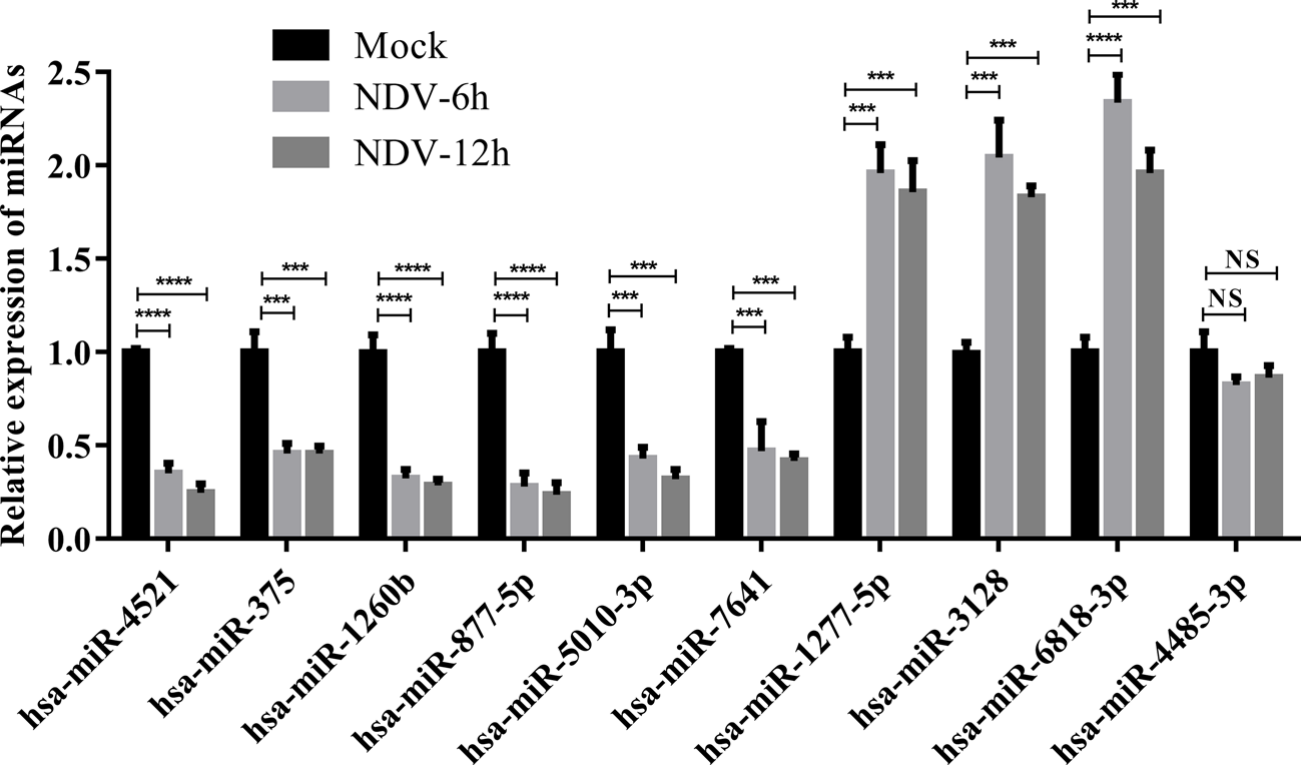
Verification for the expression patterns of 10 selected SDE miRNAs by qRT-PCR. HeLa cells were infected with Herts/33 at an MOI of 1. Total miRNAs in HeLa cells were extracted at 0, 6 and 12 hpi, respectively. Expression levels of 10 selected SDE miRNAs were measured by qRT-PCR. The relative expression level of each miRNA in the NDV-infected HeLa cells was calculated using the 2^-ΔΔCT^ method and represented as the n-fold change relative to the mock-infected sample. Bar diagrams show the means ± SD obtained from triplicate experiments. *** stands for *P*-value < 0.001, **** stands for *P*-value < 0.0001 and NS stands for non significant.

To validate the results of deep sequencing, qRT-PCR was performed on 10 miRNAs, which were randomly selected from 23 common SDE miRNAs. As shown in **Figure 6**, 6 miRNAs (hsa-miR-4521, hsa-miR-375, hsa-miR-1260b, hsa-miR-877-5p, hsa-miR-5010-3p and hsa-miR-7641) showed significantly down-regulated and 3 miRNAs (hsa-miR-1277-5p, hsa-miR-3128 and hsa-miR-6818-3p) showed significantly up-regulated in both NDV-6h and NDV-12h group when compared with mock group. In addition, hsa-miR-4485-3p showed a slight down-regulated but non-significant difference in both NDV-6h and NDV-12h group when compared with mock group. In general, the majority (9/10) of the randomly selected miRNA expression profiles by qRT-PCR were consistent with those obtained by sRNA deep sequencing.

### Hsa-miR-4521 Inhibits NDV Replication in HeLa Cells

**Figure 7:**
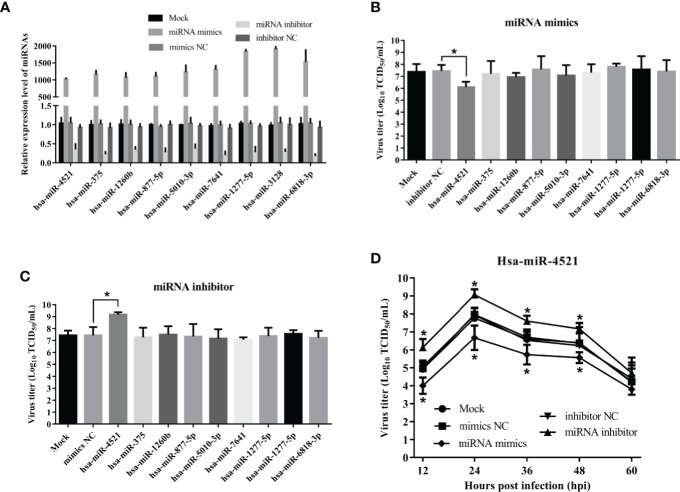
The effect of 9 verified SDE miRNAs on NDV replication in HeLa cells. **(A)** HeLa cells were transfected with indicated miRNA oligonucleotides (final concentration, 100 nM) and the expression levels of corresponding miRNAs were measured by qRT-PCR assay at 18h after transfection. **(B, C)** HeLa cells transfected with corresponding miRNA mimics **(B)** or miRNA inhibitor **(C)** were infected with Herts/33 at an MOI of 1. After 24h, viral titers in cellular supernatants were measured by TCID_50_ as described above. **(D)** HeLa cells were transfected with indicated miRNA oligonucleotides at a final concentration of 100 nM or left untreated. Eighteen hours after transfection, HeLa cells were infected with Herts/33 at an MOI of 0.1. The viral road in the supernatants collected at different time points was quantified by TCID_50_ in DF-1 cells. Data are from three independent experiments and presented as the means ± SD. * stands for *P*-value < 0.05.

MiRNAs have been implicated in regulating virus replication (21). To test if miRNAs regulated by NDV also affected NDV replication, we firstly detected the efficiency of miRNA mimics and inhibitors by qRT-PCR. As shown in **Figure 7A**, miRNA levels in HeLa cells transfected with their corresponding miRNA mimics were dramatically increased (superior to 1000 folds) but was decreased by 30% in HeLa cells transfected with miRNA antagonists. Subsequently, indicated miRNA oligonucleotides, including miRNA mimics or inhibitors or negative control, were transfected into HeLa cells followed by NDV infection and viral titers in the supernatants were quantified by TCID_50_. As a result, hsa-miR-4521 significantly decreased NDV replication in HeLa cells (**Figure 7B**), whereas hsa-miR-4521 antagonist increased NDV replication (**Figure 7C**). Virus growth curves also revealed that hsa-miR-4521 negatively regulated NDV replication (**Figure 7D**). These data indicated that hsa-miR-4521 may play an antiviral role during NDV infection in HeLa cells.

### FAM129A Is a Target of hsa-miR-4521 and Promotes NDV Replication in HeLa Cells

**Figure 8:**
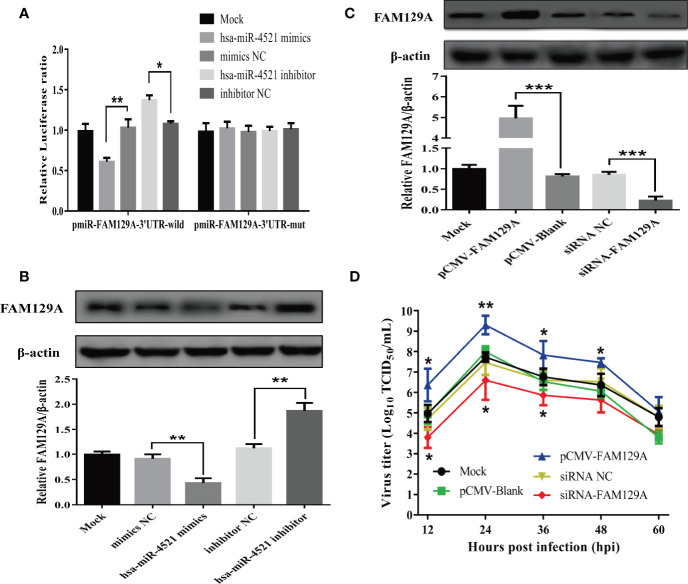
FAM129A was directly targeted by hsa-miR-4521 and positively regulated NDV replication in HeLa cells. **(A)** HeLa cells were co-transfected with indicated miRNA oligonucleotides and plasmids, and *renilla* luciferase activity was measured and normalized to firefly luciferase activity after 48h. **(B, C)** The expression of FAM129A protein in HeLa cells was detected by Western blot assay at 18h after indicated miRNA oligonucleotides **(B)** or plasmids **(C)** were transfected and the relative intensity of FAM129A was normalized to β-actin and showed in the below. **(D)** HeLa cells were transfected with indicated plasmids or siRNAs, eighteen hours after transfection, cells were infected with Herts/33 at an MOI of 1 and the viral growth curves were measured by detecting virus titers in the supernatant collected at indicated time points. Data are from three independent experiments and presented as the means ± SD. *stands for *P*-value < 0.05, **stands for *P*-value < 0.01 and *** stands for *P*-value < 0.001.

Previous study has revealed that FAM129A, also known as Niban or C1orf24, is a target of hsa-miR-4521 and acts as a promoter in clear cell renal cell carcinoma (ccRCC) (32). In order to confirm whether hsa-miR-4521 targets FAM129A in HeLa cells, hsa-miR-4521 binding site in the 3’UTR of FAM129A was predicted and cloned to generate pmiR-FAM129A-3’UTR-WT. Meanwhile, this wild-type plasmid was used as template to construct a hsa-miR-4521 binding site mutated one named pmiR-FAM129A-3’UTR-mut. Then, a luciferase reporter assay was conducted at 48h after co-transfection of indicated plasmids and miRNAs. As shown in **Figure 8A**, hsa-miR-4521 negatively regulated the relative luciferase activity in cells transfected with pmiR-FAM129A-3’UTR-WT, but not pmiR-FAM129A-3’UTR-mut, indicating that hsa-miR-4521 can directly bind to the 3’UTR of FAM129A. Moreover, western blot assay showed that overexpression of hsa-miR-4521 significantly decreased the expression of FAM129A protein, while hsa-miR-4521 inhibitor exhibited an opposite effect (**Figure 8B**). In conclusion, these results strongly revealed that hsa-miR-4521 can directly target FAM129A, thereby suppressing its translation efficiency in HeLa cells.

Finally, we determined if hsa-miR-4521 regulated NDV replication by FAM129A. HeLa cells transfected with an FAM129A expression vector or FAM129A-specifc siRNAs were infected with NDV and analyzed for virus replication. As shown in **Figure 8C**, the levels of FAM129A increased by about 5 folds in HeLa cells transfected with pCMV-FAM129A and siRNA-FAM129A down-regulated the expression of FAM129A by about 80%. Virus growth curves of NDV in FAM129A-overexpressed or –knocked down HeLa cells showed that FAM129A overexpression increased NDV replication in HeLa cells (**Figure 8D**). Conversely, FAM129A knockdown significantly inhibited virus replication.

## Discussion

MiRNAs regulates the host-pathogen interactions (33). A variety of viruses are capable of modulating the expression of cellular miRNAs to facilitate their replication. These miRNAs may reciprocally regulate virus replication. Prior studies have shown that NDV regulates miRNA expression in the cells of avian origins (34–37). NDV is an oncolytic virus and has widespread anti-tumor activities in preclinical animal models and in clinical trials (30). Our present study focuses on NDV-mediate regulation of cellular miRNA. Using high-throughput sequencing technology, we found that several dozens of cellular miRNAs were up- or down-regulated following NDV infection in HeLa cells. One of the NDV-regulated miRNAs suppressed NDV replication. Our study provides evidence that NDV replication and miRNA expression are mutually regulated by each other.

HeLa cell line was established from a human cervical cancer tissue in 1951 and has been widely used as an important tool in cancer research. This cell line has been often used for studying the oncolytic activity of a variety of viruses. We investigated miRNA expression in NDV-infected Hela cells and discovered that tens of miRNAs were differentially expressed, compared to those in mock-infected HeLa cells. Interestingly, we found that some of these miRNAs were involved in tumor progression. For example, hsa-miR-1260b is upregulated in non-small cell lung cancer (NSCLC) and promotes tumor growth, invasion, and metastasis by targeting PTPRK (Protein Tyrosine Phosphatase Receptor Type K) and SOCS6 (Suppressor of Cytokine Signaling 6) (38, 39). Hsa-miR-375 functions as a tumor suppressor or oncogene under different settings in various tumor types including gastric (40), hepatocellular (41), colorectal (42), esophageal (43)and breast tumors (44). Hsa-miR-7641, an oncogenic miRNA, decreases cell viability by inducing the expression of pro-apoptotic molecules in breast and colorectal cancers (45). Hsa-miR-320d (46, 47), hsa-miR-139-3p (48), and hsa-miR-1293 (49) are abundantly expressed in different types of tumors are associated with tumor initiation. Our present study shows that hsa-miR-1260b, hsa-miR-375, hsa-miR-7641, hsa-miR-320d, hsa-miR-139a-3p and hsa-miR-1293 were drastically downregulated in HeLa cells infected with NDV for at 6 and 12 hpi. Our study suggests that NDV-mediated oncolytic activity may be mediated in part by downregulating the expression of these miRNAs.

Using qRT-PCR, we validated that 9 of 10 miRNAs except hsa-miR-4485-3p were indeed differentially expressed in NDV-infected HeLa cells. GO analysis revealed that a large number of genes targeted by those differentially expressed miRNAs can regulate immune functions. Consistently, KEGG pathway enrichment analysis revealed that the genes regulated by those differentially expressed miRNAs are clustered in the JAK-STAT, NF-kB, and RIG-I-like receptor pathways, supporting the notion that NDV counteracts antiviral immune responses in part by regulating miRNA expression. In addition, KEGG analysis also revealed that the genes in the ErbB signaling pathway are the potential targets of SDE miRNAs. ErbB is an oncogene dysregulated in breast, lung, ovarian, prostate, and glioblastoma (50–55). ErbB expression is associated with poor prognosis, drug resistance and cancer metastasis (56). It will be interesting to find out if NDV indeed dampens ErbB signaling and contributes to its oncolytic activity.

Virus replication relies on the host cell machinery. The genes regulated by those differentially expressed miRNAs will likely impact virus replication. hsa-miR-4521, a member of tRNA-derived small RNAs (tsRNAs), is aberrantly expressed in a variety of tumors including lung cancer, breast cancer, chronic lymphocytic leukemia, and esophageal adenocarcinoma (57–60). However, whether hsa-miR-4521 affects the replication of oncolytic viruses has not been investigated. In the present study, we detected the viral titers in the supernatants of HeLa cells which were transfected with indicated miRNA oligonucleotides and then infected with NDV. The results indicated that overexpression of hsa-miR-4521 significantly suppressed NDV replication. Moreover, the growth curves also showed that hsa-miR-4521 negatively regulated NDV replication in HeLa cells. Considering that NDV infection significantly downregulated the expression of hsa-miR-4521, it’s easy to figure out the conclusion that NDV promotes its replication and oncolysis by downregulating hsa-miR-4521 in HeLa cells. Hsa-miR-4521 interacts with FAM129A and functions as a tumor suppressor in ccRCC (32). FAM129A, a molecule downstream of activating transcription factor 4 (ATF4) (61), inhibits apoptosis and promotes tumor cell migration and proliferation in various cancers (62–65). Using a dual-luciferase reporter assay, our study suggests that FAM129A is targeted by hsa-miR-4521. Since FAM129A was able to enhance NDV replication, hsa-miR-4521 may suppress NDV replication and oncolysis by targeting FAM129A. However, the exact molecular mechanism by which FAM129A effects NDV replication still needs to be further investigated.

Increasing evidence suggests that miRNAs-based therapeutic holds promise for the treatment of cancers and other diseases, such as miR-34 for treating pancreatic cancer and miR-122 for treating hepatitis (66). Meanwhile, previous studies proved that adjuvant like dichloroacetate could improve NDV-mediated oncolytic virotherapy (67). In our present study, we found that hsa-miR-4521-FAM129A axle plays a role in NDV-mediated oncolysis in HeLa cells, either inhibition of hsa-miR-4521 or overexpression of FAM129A can significantly promote viral replication. Sufficient viral replication determines the therapeutic efficacy of oncolytic virotherapy. Therefore, our findings provide a rationale for combinatorial strategies that combines hsa-miR-4521 antagonist with NDV for oncolytic virotherapy, which might be applied clinically in the near future. However, further studies are required to fully evaluate the synergistic effect of this potential adjuvant in NDV-mediated oncolysis through *in vivo* tests.

In summary, using high-throughput sequencing, the miRNA expression patterns in HeLa cells followed by NDV infection were evaluated in the present study. MiRNA expression profile analysis showed that 23 SDE miRNAs, including 3 up-regulated and 20 down-regulated, showed the same expression patterns in NDV-6h and NDV-12h group. Target prediction and functional analysis of these miRNAs suggested that they function as an important role in the host immune response. Moreover, nine of 10 selected SDE miRNAs were confirmed by qRT-PCR and functional research of these confirmed SDE miRNAs revealed that hsa-miR-4521 negatively regulates NDV replication through interaction with its target FAM129A in HeLa cells. To our knowledge, this is the first report to investigate the miRNA expression data in NDV-infected tumor cells, which will contribute to further study the oncolytic mechanism of NDV.

## Data Availability Statement

The original contributions presented in the study are publicly available. This data can be found here: https://www.ebi.ac.uk/arrayexpress/experiments/E-MTAB-9690/.

## Author Contributions

YC was responsible for experiment design, data analysis and writing the manuscript. SZ and YP were responsible for performing experiments. JH, ZH, XW, XWL, MG and SH were responsible for suggestion during the experiment’s performance. XFL was responsible for revising the manuscript. All authors contributed to the article and approved the submitted version.

## Funding

This work was funded by grants from the National Key Research and Development Program of China (2017YFD0500101-3, 2017YFD050080203), by the National Natural Science Foundation of China (31873021). The Earmarked Fund For China Agriculture Research System (CARS-40), the National Key Research and Development Program of China (2017YFD0500702), and a project funded by the Priority Academic Program Development of Jiangsu Higher Education Institutions.

## Conflict of Interest

The authors declare that the research was conducted in the absence of any commercial or financial relationships that could be construed as a potential conflict of interest.
